# Fabrication and Modeling of a Thermoreversible Modular Core–Shell Colloidal System

**DOI:** 10.1021/acs.langmuir.5c03418

**Published:** 2025-09-24

**Authors:** Florence J. Müller, Alec J. Pellicciotti, Shivaprakash Ramakrishna, Lucio Isa, Michael A. Bevan, Jan Vermant

**Affiliations:** † Department of Materials, ETH Zurich, Vladimir-Prelog-Weg 5, 8093 Zurich, Switzerland; ‡ Chemical & Biomolecular Engineering, 1466 Johns Hopkins University, Baltimore, Maryland 21218, United States; § Department of Chemical Engineering, California Institute of Technology, Pasadena, California 91125, United States

## Abstract

A widely used model system in rheological studies of colloidal gels consists of octadecyl-coated silica particles that undergo thermoreversible gelation in specific suspending media. Their standard synthesis protocol involves an etherification of octadecanol and suffers from poor reproducibility with varying grafting densities and, therefore, transition temperatures. To overcome this limitation, we present here an alternative approach using an amine–yne click-like reaction to graft octadecyl chains onto the particle surface with high fidelity. Suspended in tetradecane, these particles exhibit a reversible liquid–solid transition below 20 °C—making them ideal for comparative studies, particularly by avoiding complications in the rheological characterization due to loading history or thixotropic effects. By fine-tuning the reaction conditions, we precisely control the grafting density—and thus the gelation. The resulting interparticle interactions can be described as a superposition of temperature-dependent forces: repulsion at high temperatures, van der Waals attraction, and temperature-dependent chain–chain interactions, and their resulting potentials can be validated with AFM measurements. The accurate tuning of interparticle potentials makes this model system ideally suited for quantitative comparisons between experiments and simulations across relevant length scales.

## Graphical abstract

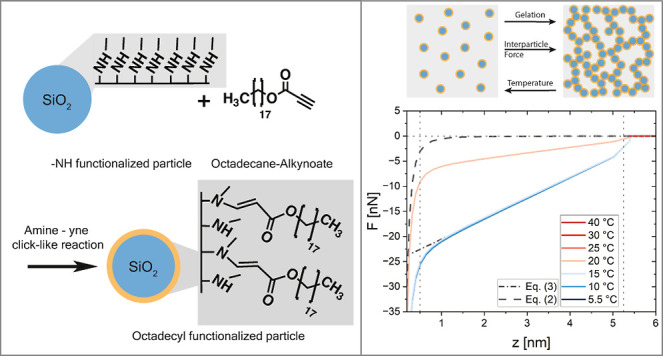

## Introduction

Colloidal gels play important roles in diverse fields, including food science (e.g., chocolate ganache) and pharmaceuticals (adjuvants in vaccines), due to their unique structural and mechanical properties. Colloidal gels are soft solids formed by a space-spanning network of weakly attractive submicron particles that lock in liquid and yield under small stresses, giving them an elastic yield point, strong shear-thinning, and rapid self-healing (thixotropic) behavior even at low solid content. In applications, colloidal gels oftentimes have a complex formulation, which makes their behavior difficult to deconvolute. Therefore, specific colloidal material properties are oftentimes studied through model systems, which allow the study of a single parameter. These model systems must closely replicate the particle features (volume fraction, size, and shape) as well as the gel structure and behavior of actual colloidal gels to ensure practical relevance. Rheological model systems, in particular, must also be compatible with the operating windows of modern rotational rheometers.^1,2^ Moreover, colloidal gels are typically highly thixotropic, which means that their properties depend on the shear history;^3,4^ therefore, loading colloidal gels into the rheometer can again bias the experiment and reduce reproducibility.^5,6^ One way to circumvent this effect is to use a thermoreversible sample, which can be gelled and fluidified through external temperature changes.^7^ Ideally, the sample is loaded in a liquid state followed by gelation, through a specific protocol (e.g., change in the temperature) that ensures reproducible kinetic pathways, inside the measurement geometry.^8^

Many of the criteria mentioned above can be met with the model system of silica particles, coated with octadecyl, which are suspended in tetradecane. In particular for these systems, (i) silane chemistry is extremely versatile, allowing for the fabrication of particles with different shapes and topographies.^16–18^ (ii) The suspending media tetradecane is nonvolatile, which reduces evaporation effects. (iii) Thixotropic effects can be circumvented through the thermoreversible nature of the suspension–the system is a gel at low temperatures and liquid at high temperatures.^8^ However, achieving a reproducible and straightforward synthesis remains a major challenge when evaluating experimental protocols. While surface grafting of silica particles with oligomers and polymers is a widely adopted technique, it is often difficult to reproduce and may require inert conditions or be limited to very small batch sizes.

In 1981, Van Helden et al. published a much used synthesis method, where Stöber silica particles^19^ could be functionalized with octadecyl in large batches^7^ using a direct etherification of octadecanol onto the hydroxyl surface groups of the silica particles (see Figure 1a). This reaction is usually performed with a large excess of octadecanol in order to maximize the grafting density of the particles. However, looking at the literature that used this synthesis over the past decades, there is a substantial discrepancy of achievable grafting densities which is reflected in the variations of the gelation (liquid-to-solid) temperature^15^ (see Figure 1b). In the original publication detailing the grafting process, Van Helden et al. report a grafting density of 0.15–0.23 chains/nm^2^,^7^ which was characterized using thermogravimetric analysis. Later, Eberle et al. report a much higher grafting density of 2.4 chains/nm^2^, measured using neutron reflectometry.^8^ Another existing method to graft octadecyl to the surface of silica particles is through silane chemistry (hydrolysis followed by the condensation of two hydroxyl groups) of trimethoxy (octadecyl)­silane (see Figure 2a); however, these systems do not show a temperature-dependent liquid-to-solid transition^20,21^ (see Figure 2b.). This is likely due to the distribution of the grafting agent on the particle, which is not homogeneous but patchy (see Figure 2c).

**Figure 1:**
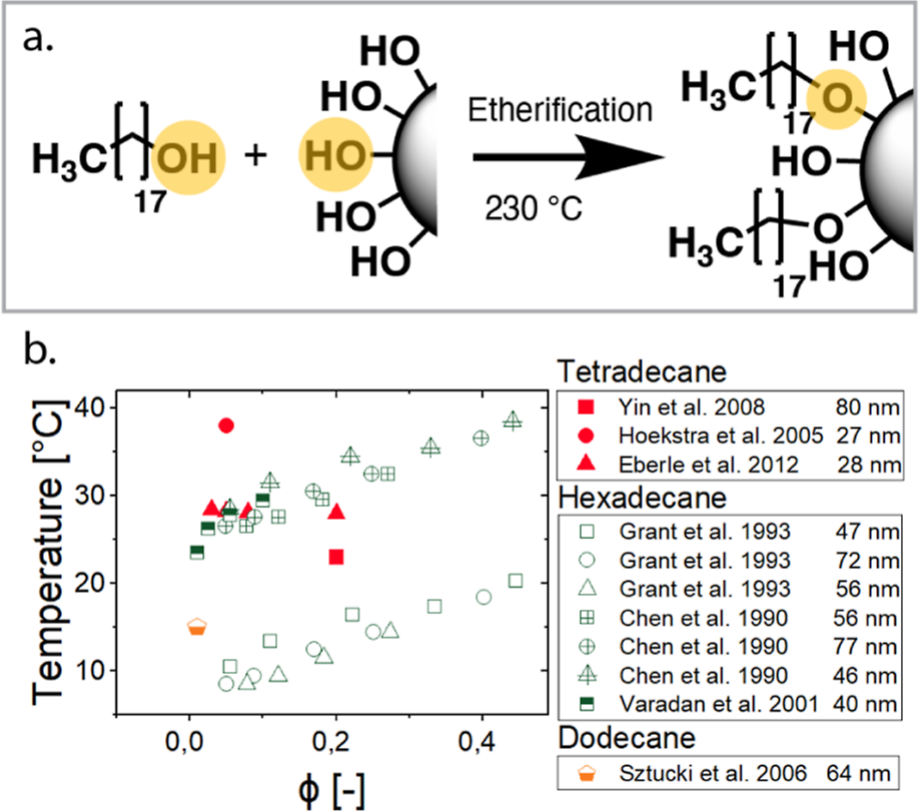
Literature values of the gel transition for octadecyl-coated silica particles synthesized by the Van Helden method. (a) Direct etherification of octadecyl onto the silica particle (b) Literature values of transition temperatures, gels are formed in different suspending media: (i) tetradecane: Yin and Solomon 2008,^9^ Hoekstra et al. 2005,^10^ Eberle et al. 2012;^11^ (ii) hexadecane: Grant and Russel 1993,^12^ Chen and Russel 1990,^13^ and Varadan and Solomon 2001;^14^ and (iii) dodecane: Sztucki et al. 2006.^15^ The diameter of the particles is indicated on the right of the legend in nm.

**Figure 2:**
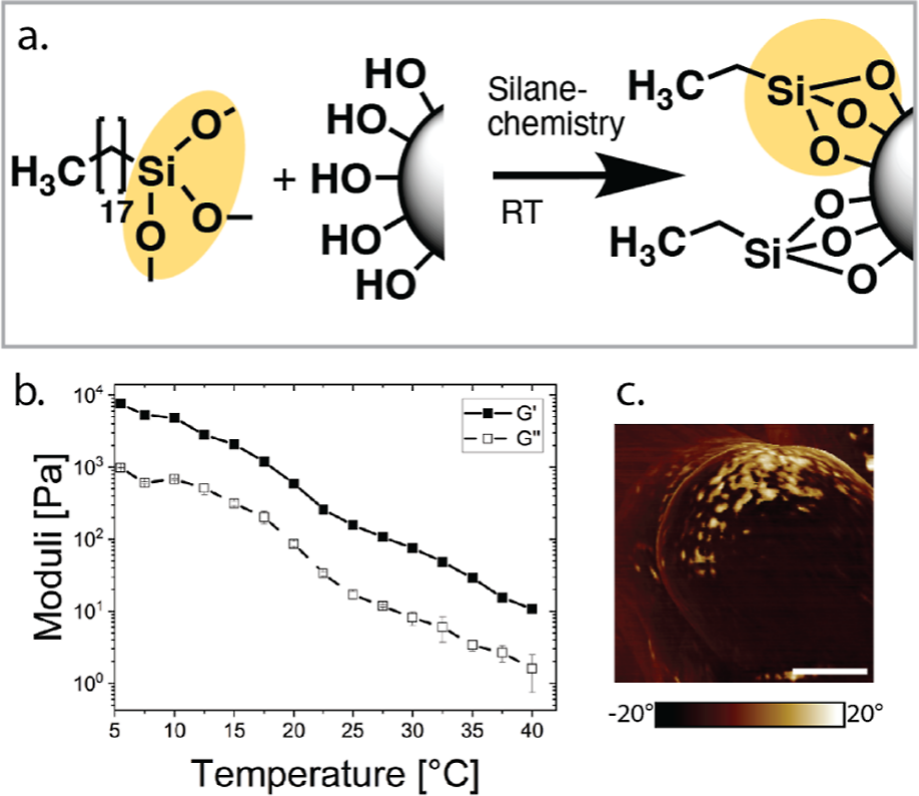
Grafting octadecyl using silane chemistry (a). Grafting octadecyl silane to the surface of Stöber particles; (b) temperature sweep of a gel (ϕ = 0.25) of octadecyl particles grafted with octadecyl through silane chemistry in tetradecane; and (c) AFM image of the particles using phase imaging, where the bright color represents the octadecyl patches and the dark color represents the silica substrate, reproduced from ref 22 Copyright 2025 American Chemical Society.

While these approaches have been successful in generating particles for colloidal gels, the variability mentioned above presents a substantial challenge for the development of robust and reproducible gels. In fact, a key question in the field of colloidal gels is understanding how the network microstructure correlates with macroscopic gel properties.^23,24^ Achieving this requires a comprehensive understanding of the physics at the level of particle pairwise interactions and at the cluster level, which can be achieved through computational and mathematical models that complement experimental findings.^25–27^ A prerequisite for these models are a precise definition of the separation distance dependent interparticle potential.^28^ In depletion gels—where particle aggregation is driven by osmotic pressure from the added depletants—potentials like the Morse potential^29^ or those based on the Asakura–Oosawa model^30^ are often used to describe interparticle forces. These potentials enable computational models that closely approximate physical systems.^31^ For thermoreversible particle stabilization, however, open questions remain about quantifying adhesive interactions, particularly for colloids stabilized by shorter ligands in index-matched media.

In thermoreversible polymer-stabilized colloidal dispersions, polymers on the surfaces of the particles are often responsible for gelation. Whether interactions in polymer-stabilized colloidal systems are attractive or repulsive can be explained by the solvent quality. “Good” solvent conditions result in minimal van der Waals attraction from the polymer layer due to the similarity in the dielectric properties of the solvent medium and highly solvated polymer layers. On the other hand, “poor” solvent conditions increase van der Waals attraction due to the densification of the chain structure and resulting dissimilarity in the dielectric properties between the desolvated polymer layer and solvent medium.^32^ The temperature can switch a given solvent species between a “good” and “poor” solvent (through the well-known “theta temperature”),^33^ and hence modify interparticle forces.

However, for colloids stabilized by short ligands in index matched media, the van der Waals is minimized, and it has been shown that the difference in chain density and solvent fraction upon crystallization at low temperatures is minimal.^8^ This likely results in only a relatively small change in the van der Waals attraction due to chain density increases and the associated contrast in ligand-solvent dielectric properties.

In addition, when considering the small temperature dependence of alkane dielectric properties,^34,35^ there is not an obvious temperature-dependent van der Waals attraction mechanism to explain the onset of gelation in colloid-ligand systems. Therefore, it has been speculated that the temperature-dependent phase behavior of octadecyl-stabilized colloidal particles must depend on other attractive interparticle forces in addition to van der Waals forces.^34–36^ Supporting this conclusion, Jansen et al. found that van der Waals interactions alone (based on approximate models) could not account for the observed aggregation of octadecyl-silica spheres and instead employed a square-well potential with well depth estimated using the Flory-Krigbaum theory, to describe the temperature-dependent phase behavior.^11,37^

To address the issues of experimental reproducibility and direct mapping onto quantitative models, the present work proposes a streamlined synthesis approach, which is more controllable, reproducible, and highly modular using amine-yne click-like chemistry. This chemistry has been used in the literature for an in situ two-component hydrogel system^38^ and as an efficient polymerization approach for poly­(enamine)­s.^39^ We synthesize octadecyl-grafted particles in a multistep process (see Figure 3), which allows for a quantitative comparison of different batches, making it appealing as a rheological model system. Varying the reactant stoichiometry allows for the tuning of the grafting density and, therefore, the temperature of the liquid-to-gel transition. We quantitatively compare particle interaction forces measured through colloidal probe AFM with theoretical predictions and show that we can accurately model the interactions and phase behavior of octadecyl-grafted colloids—and, more generally, any short-ligand system—which requires the inclusion of additional attractive forces. To that end, we propose a novel temperature-dependent chain–chain potential that describes ligand–ligand interactions between neighboring particles or surfaces, which accounts for additional attraction that is insufficiently described by van der Waals forces between solidified ligand layers (based on rigorous calculations via the Lifshitz theory). This work presents a modular reproducible model system that enables reliable rheological measurements and direct comparison with simulations through a well-defined and physical interparticle potential.

**Figure 3:**
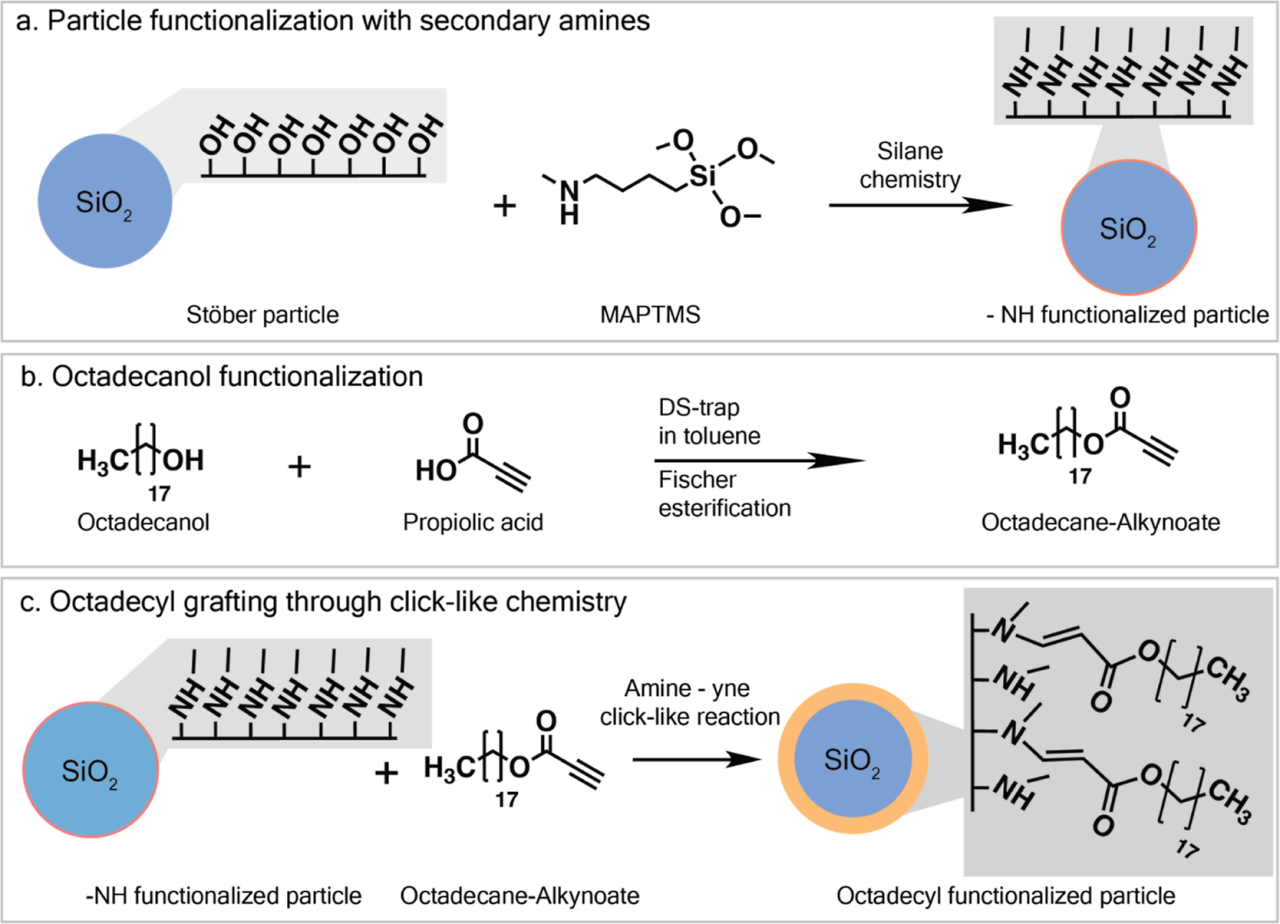
Multistep synthesis approach for octadecyl-grafted silica particles using amine-yne click-like chemistry. (a) Functionalization of silica particles through hydrolysis and condensation of the silanol groups. (b) Functionalization of octadecanol with an alkynoate group through Fischer esterification. (c) Amine-yne click-like reaction to graft the octadecane-alkynoate to the secondary amine groups on the surface of the silica particles.

## Materials and Methods

### Synthesis

#### Materials

Ethanol (99.8%, Merck), tetraethyl orthosilicate (TEOS, 99%, Sigma-Aldrich), ammonia solution (NH_4_OH, 25%, Merck), triethyl­(octadecyl)­silane (TMOS, Sigma Aldrich), trimethoxy­[3-(methylamino)­propyl]­silane (MAPTMS, 97%, Sigma-Aldrich), 1-octadecanol (Octadecanol, 99%, Sigma-Aldrich) ε-caprolactone (97%, Merck Millipore), toluene (99.85%, Fisher Scientific), toluenesulfonic acid-p monohydrate (pTsOH, 98%, Sigma-Aldrich), propiolic acid (96%, Sigma-Aldrich), isopropanol (technical grade), and deionized water (Milli-Q, Merck-Millipore). All products were used as received.

#### Particle Synthesis

Silica particles (300 nm diameter) were synthesized using the Stöber process.^19^ In a typical synthesis, 200 mL of ethanol, 18 mL of MiliQ, and 10 mL of ammonia were stirred in a 500 mL glass bottle at 500 rpm. 12.4 mL of TEOS were added quickly to the solution, which was then left to react for 24 h. 3.1 mL of MAPTMS solution (5 v % in ethanol) was then added to the suspension (without a cleaning step) using a syringe pump (2 mL/h) and left to react for 1 h after the end of the injection. The particles were then cleaned 1× with ethanol and 2× with isopropanol with centrifugation and redispersion steps. Patchy particles were synthesized by adding TMOS (5 v % in ethanol) instead of the MAPTMS, these particles were then directly cleaned and dried without the click-like reaction.

#### Octadecane-Alkynoate Functionalization

The octadecanol was functionalized with an alkynoate group following the Fischer esterification process. For this, 10 g (1 equiv) of octadecanol was dissolved in 70 mL of toluene at 50 °C in a 100 mL round-bottom flask. After complete dissolution, 0.5 g (5% of octadecanol weight) of pTsOH was added to the reaction, followed by 2.52 mL (1.1 equiv) of propiolic acid. A Dean–Stark trap and a condenser were then installed on the round-bottom flask, and the solution was heated to 135 °C to start the reaction, which was left for 24 h. The octadecane-alkynoate was purified by evaporating the toluene, and the product was then dissolved in 20 mL of acetone and dropped into iced Mili-Q for the octadecane-alkynoate to precipitate. The suspension was then isolated using a vacuum filter and dried under a vacuum at 30 °C for 24 h. The product was stored in a freezer to avoid degradation.

#### Amine-yne Click-Like-reaction

The octadecane-alkynoate was dissolved in isopropanol at 5 wt % at 40 °C. To graft 1 g of secondary amine-functionalized core particles, 1.25 mL of the octadecane-alkynoate stock solution was added to a 10 wt % suspension of particles in isopropanol at 40 °C and left to stir for 3 h. The functionalized particles were then washed with isopropanol three times and dried in a rotary evaporator and subsequently in a vacuum oven for 48 h.

#### Gel Formation

The dry octadecyl-functionalized particles were suspended in tetradecane and heated to 60 °C for 20 min in a water bath. In order to ensure the complete dispersion of the particles, the suspension was tip-sonicated for 10 s to break up aggregates.

### Characterization

#### TGA

TGA experiments were performed with a TA Instruments TGA 5500. Approximately 10 mg of the dried sample was measured after taring the crucible. The program first equilibrated at 30 °C and then ramped to 900 °C at 5.00 °C/min.

#### X-ray Photoelectron Spectroscopy (XPS) Analysis

The elemental composition of the sample surface was analyzed by X-ray photoelectron spectroscopy (XPS, AXIS UltraDLD). An Al target X-ray source with a monochromator (Al Ka, *h*ν = 1486.7 eV) was used, and the pass energy was 50 eV. The XPS spectra were analyzed with XPSPEAK Version 4.1. All binding energies are referenced relative to the hydrocarbon peak at a binding energy of 285.0 eV. All the peaks were analyzed with the Gaussian–Lorentzian curve-fitting in the software CASAXPS (80% fraction of Gaussian character).^40^

#### Atomic Force Microscopy (AFM)

The characterization of the grafted layer through AFM was carried out by a Bruker Dimension Icon AFM. The measurements were conducted by immersing the octadecyl functionalized silicon wafers in a custom-made temperature cell^22^ containing tetradecane. The temperature was gradually increased from 5.5 to 40 °C in an interval of 5 °C using the heater-cooler system. Before each measurements, the temperature was stabilized for 20 min and maintained an accuracy of ±1 °C. In the ligand layer height experiments (shown in Supporting Information, Figure S7), a part of the surface was scratched with tweezers, to remove the grafted layer and the height was measured across the scratched surface and the remaining ligand layer.

#### Rheology Measurements

Rheology measurements were performed on an Anton Paar MCR 502 using a 20 mm plate–plate geometry with a pillar roughness of 100 μm on the top and bottom. The sample was loaded at 60 °C, cooled down to 5 °C, and equilibrated for 30 min. Between the measurements, the gel was rejuvenated by cycling the temperature to 60 °C for 20 min and cooled to 5 °C for 30 min. All measurements were performed at 5 °C. No significant influence of the cooling rate was observed, in all experiments the temperature was decreased in a single step; however, cooling itself was slow, due to the thermal inertia of the Peltier element, the rough bottom plate, and the sample itself. The stress amplitude sweep measurements were performed at 1 rad/s. The continuous creep measurements were performed for 5 min. In the recovery measurements, the sample was sheared at 100% strain and 1 rad/s for 1 min before letting the structure recover at a strain of 0.01%. The time scales of these experiment were chosen so that they will not be affected by physical^41^ or physicochemical^42^ aging effects (40 min for temperature equilibration +1000 s for reliable data acquisition without aging effects).

#### Rheoconfocal Measurements

Rheoconfocal measurements were performed using a previously developed custom setup,^43^ where the lower plate consists of a glass slide through which a confocal microscope scans through the sample in the *z*-direction. The temperature was controlled by using a commercially available Peltier hood (Peltier Temperature Device 200, Anton Paar) through a heated or cooled nitrogen stream on the upper geometry (20 mm with a pillar roughness of 100 m). The glass slide that served as a lower geometry plate was functionalized with octadecyl, in order to reduce slip during the measurement.

### Interparticle Interactions

The grafted octadecyl silica system was modeled a core–shell particle with a silica core and a uniform octadecyl film. The interaction potential of octadecyl silica particles were modeled from the superposition of hard wall repulsion, van der Waals attraction, and subsequently additional temperature-dependent attraction, which considers chain–chain interactions. The Lifshitz theory was used to compute the separation-dependent Hamaker function in the van der Waals potential for two different cases: overlapping octadecyl chains and nonoverlapping octadecyl chains. In the case that more information on the octadecyl layer is available, a continuous transition between the two states can be computed, following the approaches of Bevan and Prieve 1999,^44^ Dagastine, Bevan, White, Prieve 2004,^45^ and Bevan, Petris, Chan 2002.^32^ The Derjaguin approximation for a sphere and a half-space was applied to each Hamaker function to obtain two geometrically corrected van der Waals potentials, where a single power law was fit to the potential for nonoverlapping chains at large separations and the potential for overlapping chains at separations approaching contact (Supporting Information, Section S3). The temperature-dependent attractive interchain potential was tuned using the parameter Λ to fit the superposition of forces at silica–silica separations of *z* = 5.35 nm and *z* = 0.5 nm with the forces measured by AFM during the approach and retraction of the probe, respectively. For convenience, we present the negative of the forces measured by AFM for the retraction of the probe. This fit was performed at each temperature (5.5, 10, 15, 20, 25, 30, and 40 °C), then a sigmoid was fit to the values of Λ to obtain a temperature-dependent function of the attractive interaction potential.

## Results

### Experimental Model System

Octadecyl-functionalized silica particles are fabricated in a multistep process (see Figure 3). Silica particles are synthesized using the Stöber method^19^ and functionalized with secondary amines using silane chemistry (see Figure 3a). In parallel, octadecanol can be modified with a functional alkynoate group through Fischer esterification (Figure 3b). The particles are then finally grafted with the oligomer through a click-like reaction between the secondary amine on the particle and the alkynoate group at the end of the octadecyl by simple mixing of the components in isopropanol at 40 °C (see Figure 3c and detailed approach in Supporting Information, Section S1).

The thermoreversibility of this colloidal gel model system allows circumventing thixotropic effects during the rheological characterization of the gel such as shear-induced densification, heterogeneity and anisotropy as possible outcomes.^46^ Typically, gel preparation starts from a powder in its suspending medium, followed by vortexing, sonication, and stirring. The sample is then loaded into the measurement cell (e.g., rheometer plates) using a spatula or a pipette. All these processes are extremely ill defined, involve high shear rates, and can be operator dependent, inducing sample-to-sample variability. Therefore, the use of a thermoreversible model system greatly improves the reproducibility of comparative studies. Figure 4a shows that cycling the temperature between 15 and 40 °C will form a gel and fluidify the sample. This cycle can be repeated numerous times until the particles are affected by gravitation-induced sedimentation. The plot in Figure 4a shows that the sample needs about 20 min for the temperature to equilibrate and the gel to form. When the temperature is increased, the linear viscoelastic moduli drop and *G*″ overtakes *G*′ (here shown only at one frequency), which is an indication of the fluidification of the sample. Figure 4b shows the microstructure of the gel using confocal imaging in a rheometer, after loading of the gel (at 5 °C) using a pipette and squeezing the sample to accurately fill the rheometer geometry. The microstructure shows a heterogeneous structure, with some dense and some particle-depleted regions, most likely due to deformations during sample loading. When the structure is heated to 40 °C (Figure 4c), the particles are repulsive and, therefore, homogeneously distributed throughout the field of view. Then, the sample can be cooled down to 5 °C again to induce gelation, and a much more homogeneous network structure is obtained using this thermal pathway (see Figure 4d). In these experiments, no significant influence of the cooling rate was observed. Using the click-like chemistry approach allows for a homogeneous coating of the grafting agent, which was shown through phase-contrast AFM measurements on a particle surface (see Figure 4e). The image clearly shows a much more uniform surface compared to the AFM image, showing a patchy octadecyl coverage when using the silane chemistry approach (see Figure 2c). The modularity of this approach further allows for the grafting with other ligands, oligomers, and polymers such as tetradecanol (see Supporting Information, Figure S3), eicosanol (see Supporting Information, Figure S4), polyethylene glycol (see Supporting Information, Figure S5), and polyethylene glycol-*block*-polycaprolactone (see Supporting Information, Figure S6). To that end, the ligand in question can be functionalized with an alkynoate group analogously to the octadecanol and grafted to the particles through mere mixing.

**Figure 4:**
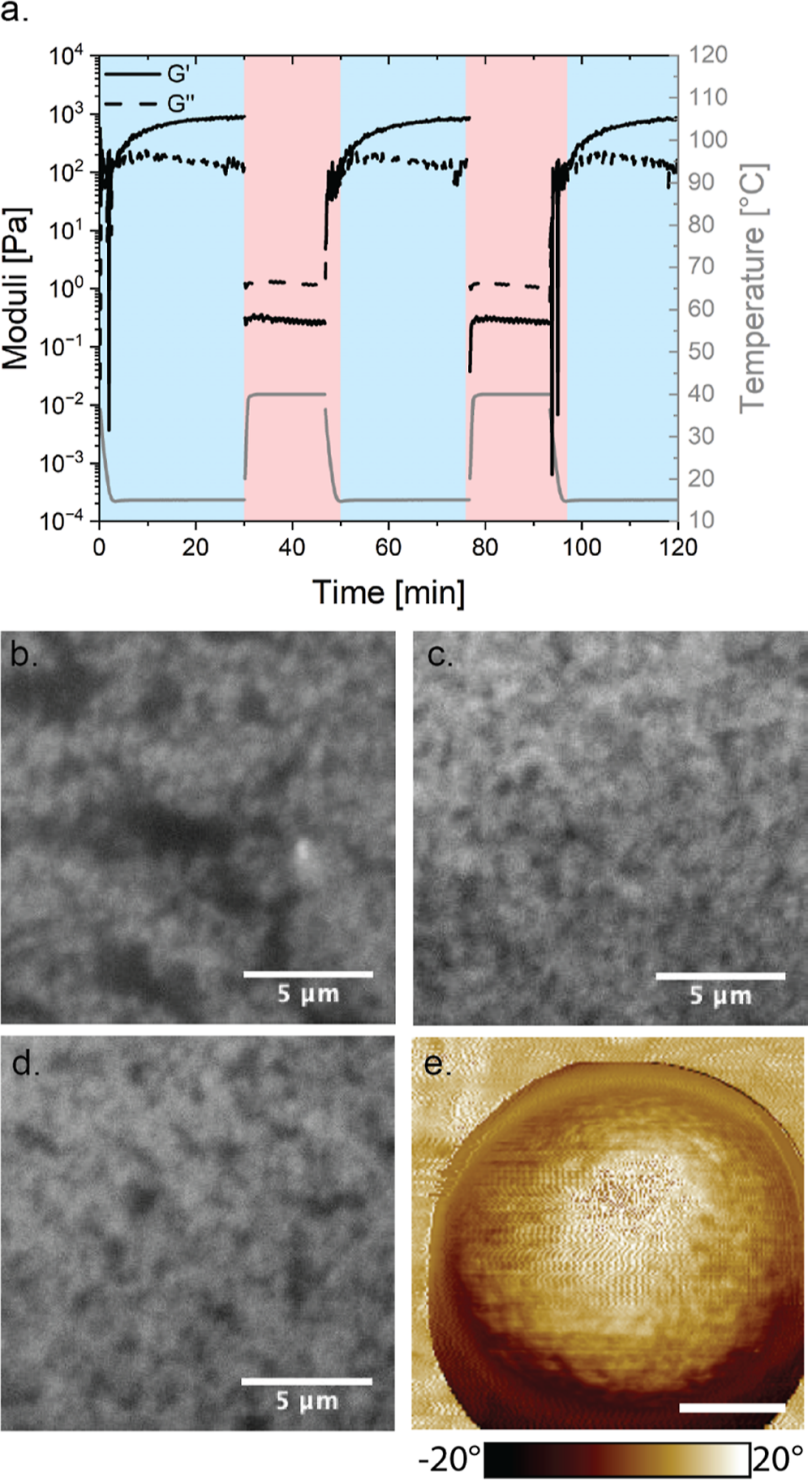
Thermoreversibility of the octadecyl-grafted silica particles (300 nm in diameter) in tetradecane (ϕ = 0.25). (a) Temperature cycling between 15 and 40 °C to gel and fluidify the structure. (b) Microstructure of the gel after loading using a pipette at 5 °C. (c) Microstructure of the particle suspension after heating to 40 °C, (d) microstructure of the gel after cooling to 5 °C, and (e) phase contrast AFM image of the silica particle coated with octadecyl, where a positive 20° phase angle indicates the stiffness of the octadecyl and a negative 20° phase angle would indicate the harder material of SiO_2_ (scale bar = 100 nm), reproduced from ref 22, Copyright 2025 American Chemical Society.

### Tuning the Grafting Density

The grafting density, meaning the amount of grafting agent per surface area, of the particles can be tuned quite easily through the reaction conditions. Different grafting densities are achieved by varying the stoichiometric ratios of the secondary amine groups and the octadecane-alkynoate. We employed both thermogravimetric analysis (TGA) and X-ray photoelectron spectroscopy (XPS) to estimate the amount of octadecyl on the particles. In this experiment, we synthesized one large batch of amine-grafted silica particles (after keeping 50 mg as a control) and split into 4 different batches which were all grafted with different amounts of octadecyl. Therefore, the relative mass of the secondary amine should be equivalent in all samples. Figure 5a shows the TGA experiments, where the sample is heated to 900 °C and the weight loss is monitored. Octadecanol has a flaming temperature of 210 °C, and the curves representing the octadecyl-grafted silica particles show a clear drop in mass around that temperature. All samples show a decrease in the mass between 150 and 500 °C, temperatures associated with the degradation of organic compounds, such as amine and hydroxyl surface groups. At temperatures above 500 °C, the silica begins to undergo calcination, which means that unreacted silanol groups inside the particle continue to react, which releases water and leads to further loss of the sample’s mass.^47,48^ The grafting density of the octadecyl-grafted samples was calculated using this difference in relative mass reduction from TGA at 900 °C.

**Figure 5:**
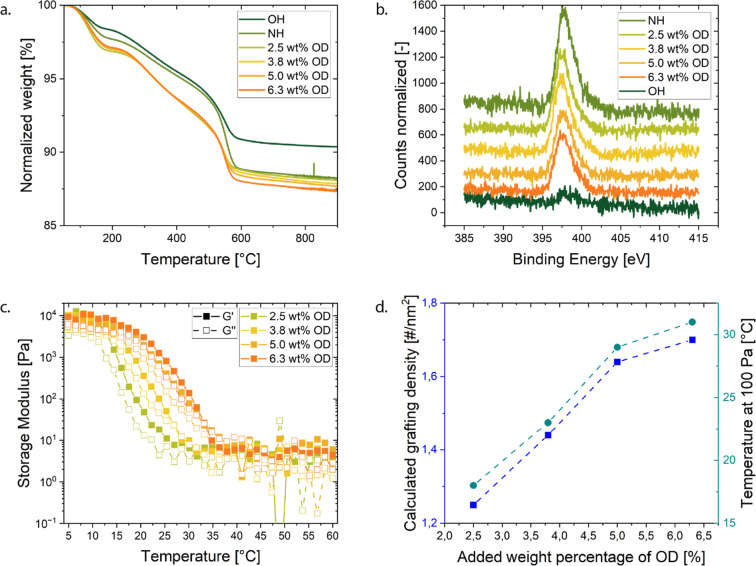
Characterization of the grafting density. (a) TGA measurement of silica particles before functionalization (OH), with secondary amines (NH), and with four different grafting densities through the variation of the added weight percentage of octadecyl-alkynoate to the click-like reaction. (b) XPS spectrum of the binding energy for secondary amines. (c) Temperature ramp of a 25 v % gel in tetradecane for the different grafting densities of octadecyl on silica. (d) Grafting density calculated from the TGA and the temperature at which the gel surpasses the elastic modulus of 100 Pa in the temperature sweep from a gel at 25 v %.

Figure 5b shows the corresponding XPS characterization of the same samples as for the TGA analysis above. The amine peaks clearly show that the control group that has not been grafted (dark green) does not have a signal for the typical amine binding energy. The amine-grafted particles (without octadecyl coating) show the largest secondary amine peak, and as the grafting density increases, the amine peak decreases as the surface is increasingly covered with octadecyl.

The grafting density influences the liquid-to-solid transition temperature in tetradecane suspensions.^15^ Figure 5c shows the storage and loss moduli for various grafting densities. Ideally, the true frequency-independent gel point would be identified by a clear crossover of *G*′ and *G*″, following the Winter–Chambon criterion.^49^ In practice, these measurements were constrained by the low values of the moduli in the liquid state, which approach the instrument’s torque limit and lead to an increased noise at high temperatures. Additionally, the cooling rates in the rheometer are slow, leading to a possible interplay of gelation and cooling kinetics. Moreover, after the gel point, we are only able to access a weak frequency dependence within the accessible range of a cone-and-plate rheometer. Therefore, in Figure 5d, the transition point is instead defined as the temperature at which the elastic modulus first exceeds 100 Pa. Grafting densities, tuned between 1.25 and 1.7 chains/nm^2^ (from TGA analysis in Supporting Information, Section S2), exceed those reported by Van Helden et al. (0.15–0.23 chains/nm^2^)^7,50^ but are lower than the values reported by Eberle et al. (2.4 chains/nm^2^).^8^ Higher grafting densities correlate with higher transition temperatures, which is consistent with the gelation observations by Eberle et al.,^8^ in which they speculate that denser ligand layers interact differently at elevated temperatures. Within this particle platform, the transition temperature can, therefore, be tuned by adjusting the grafting density. Moreover, Figure 1b demonstrates that both volume fraction and particle size modulate the gel point. The volume fraction dependence reflects the percolation threshold: at low volume fractions, stronger pairwise attractions are required for gelation. Particle size impacts gelation through the size dependence of the colloidal potential and the cumulative interfacial area, which grows with the particle size at a fixed volume fraction. In this study, the particle size and volume fraction were held constant to isolate the effect of grafting density.

### Interparticle Interactions

The net separation-dependent interaction potential and forces between octadecyl-grafted silica particles dispersed in tetradecane are modeled by the superposition of hard-wall repulsion (*F*_hw_), van der Waals attraction (*F*_vdw_), and an additional attractive interaction attributed to octadecyl chain–chain interactions (*F*_cc_) between neighboring particles or surfaces

1 $$F_{tot}=F_{hw}+F_{vdw}+F_{cc}.$$

The forces are presented here to facilitate comparison with experimental AFM measurements; the corresponding potentials from which they are derived are provided in the Supporting Information, Section S3. Van der Waals attraction is modeled using the Derjaguin approximation for a particle interacting with a wall, which can further be approximated as a simple power law (see Supporting Information, eq S7), whose derivative with respect to the separation distance is the van der Waals force such that

2 $$F_{vdw}=-p\left(A\left(z\right)\times a/6\right)z^{-p-1},$$

where *a* is the particle radius, *p* is a fitting parameter determined by a least-squares minimization with the potential (*U*_vdw_), *z* is the silica–silica separation distance, and *A*(*z*) is a separation-dependent Hamaker function computed from the Lifshitz theory (see Supporting Information, Section S3 for details). The temperature-independent van der Waals attraction is insufficient to account for the attractive forces measured by AFM (Figure 6c). Therefore, an additional force is added, denoted *F*_cc_, which accounts for the specific chain–chain interactions between octadecyl ligands of neighboring particles or surfaces, modeled as

3 $$F_{cc}=\Lambda \pi \left(2a+z\right)\left(2\delta -z\right),$$

where δ is the thickness of the octadecyl-grafted chains and Λ is a temperature-dependent parameter that controls the strength of the attraction. The remaining functional form of eq 3 arises from the overlapping volume of the plano-convex geometry formed by the intersecting spherical layers (see the Figure 6 inset in parts b and d). Λ was fit to the AFM measurements using the form

4 $$\Lambda =-B{\left(\exp\left(T-T_{C}\right)+1\right)}^{-1},$$

where *B* and *T*_c_ are adjustable fitting parameters. The results of the model provided in eq 1 as well as comparisons with the AFM data are presented in Figure 6.

**Figure 6:**
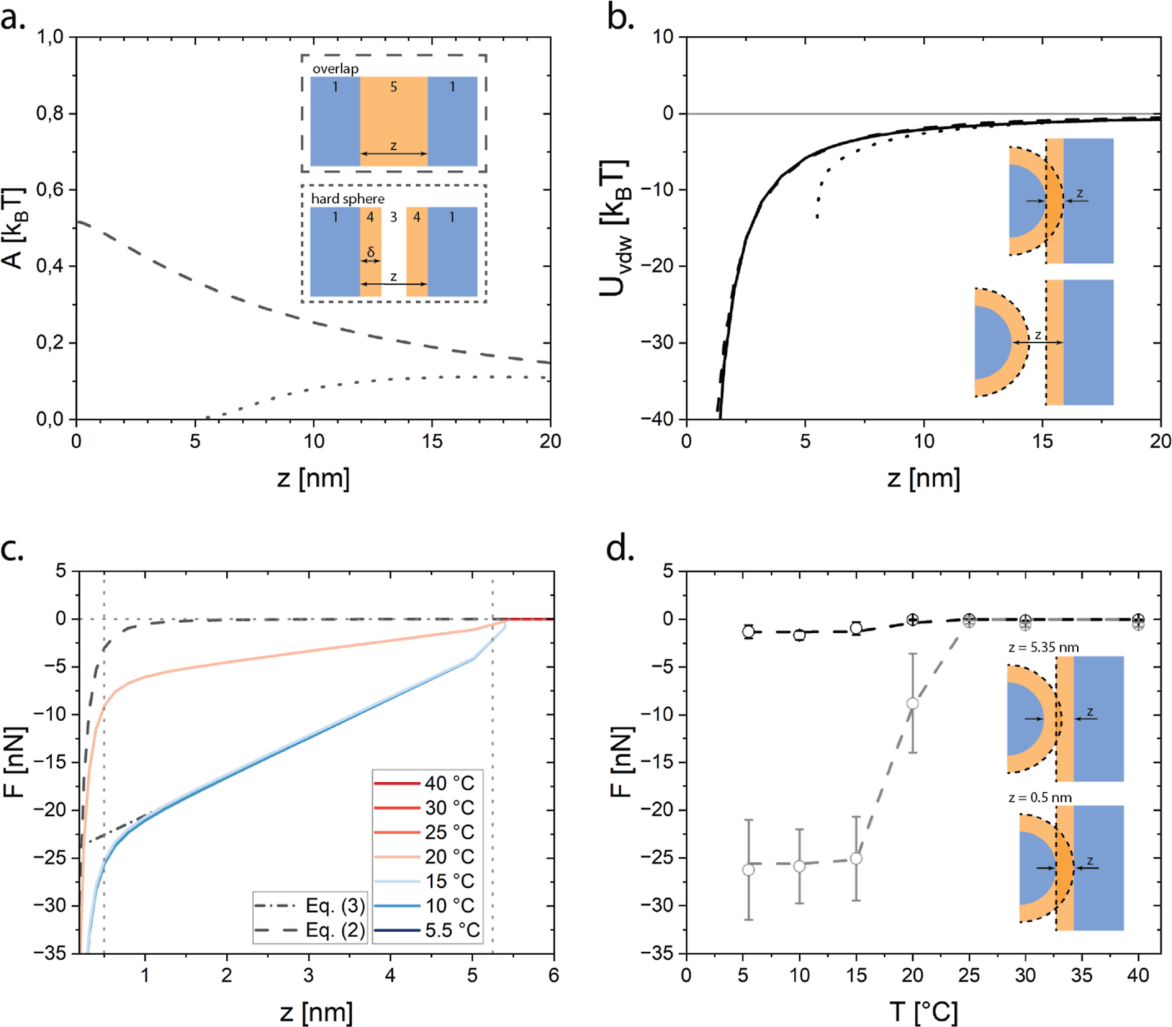
Van der Waals potential computed via the Lifshitz theory and model comparison with AFM measurements. (a) The Hamaker functions for half-spaces interacting across a medium as computed by the Lifshitz theory, using eq S11 and parameters from Table S1 in the Supporting Information. The layered system is offset by 2δ so that each function is referenced by the silica–silica separation distance (*z*). Insets show the layered system for which the Hamaker functions are computed; (b) van der Waals potential computed from the Derjaguin approximation between a sphere and a wall using the Hamaker functions in (a) with a power law fit (black line) and (c) force components (eq 2 (−), eq 3 at *T* = 278 K (.-)) and the resulting temperature-dependent superposition (eq 1) at 5.5, 10, 15, 20, 25, 30, and 40 °C. The vertical dashed lines represent separation distances used to extract forces for comparison with atomic force microscopy (AFM) measurements at *z* = 5.25 nm and *z* = 0.5 nm; (d) comparison of the model potential with AFM measurements as a function of the temperature. Both measured forces for the approach (black circles) and retraction (gray circles) of the AFM probes are presented with error bars corresponding to one standard deviation. Dashed lines represent forces extracted from (c) at *z* = 5.35 nm (black) and *z* = 0.5 nm (gray).

The Hamaker functions contributing to van der Waals attraction are computed from the Lifshitz theory for two different cases (Figure 6a,b). The first case considers the overlap of octadecyl chains between neighboring particles, modeled as silica half-spaces across a mixture of octadecane and tetradecane, with the ratio specified in Table 1. The second case considers an octadecyl-silica particle interacting as a hard sphere in a tetradecane medium, modeled as silica half-spaces with uniform octadecane/tetradecane layers across tetradecane. To ensure that the separation distance is consistent between both systems, the Hamaker function for the second case is offset by 2δ, so the separation between surfaces is defined as the silica–silica separation distance, *z*. The van der Waals potential for a particle interacting with a half-space is computed for each Hamaker function (Figure 6b), so as to compare with AFM data and fit to a power law.

**Table 1:** Parameters Used to Model Particle-Wall Interactions in Thermoreversible Colloidal Silica Gels by Eq 1

parameter	value
δ (nm)^a^	2.7
*a* (nm)	375
*p* ^b^	1.27
*B* (kT/nm^3^)	0.47
*T*_c_ (K)	262
grafting density of *C*_18_ ^c^	1.7 chains/nm^2^
surface concentration of *C*_14_ ^d^	3.1 chains/nm^2^
ϕ_C_18__	0.35
ϕ_C_14__	0.65

a C–C bond lengths.

b Obtained by least-squares minimization.

c Determined by TGA measurements.

d Estimated via Whitesides.^51^

Despite the rigorous modeling of van der Waals forces via the Lifshitz theory, considering only the temperature-dependent superposition of the forces (eq 1) combined with the individual force components (van der Waals, eq 2 and chain–chain attraction, eq 3) demonstrates that van der Waals attraction plays a relatively insignificant role in mediating attraction at distances approaching contact in the octadecyl-silica system, i.e., not allowing for overlap (Figure 6c). The weak temperature-independent attraction predicted by the Lifshitz theory aligns with high-temperature colloidal stability but cannot account for the observed temperature-dependent interactions in this system. In contrast, polymer-stabilized dispersions exhibit temperature-dependent van der Waals attractions due to conformational changes in macromolecular stabilizers driven by solvent quality.^32^ The onset of chain–chain interactions begins at 2δ, where the overlap volume between neighboring chains is nonzero and is the dominant component of attraction at small separation scales. As the temperature increases, the strength of the chain–chain interaction decreases as mediated by Λ, and shown in the fits to the AFM data in Figure 6d. To completely capture the experimentally observed temperature transition, hard wall repulsion is modeled as a piecewise function of the temperature. At T ≥ 25 °C, hard wall repulsion is applied at *z* ≤ 2δ to offset chain–chain attractive interactions, consistent with AFM measurements. At *T* < 25 °C, hard wall repulsion is applied at *z* ≤ 0 to allow chain–chain interactions near contact to be significant. AFM measurements exhibited a clear temperature transition between 20 and 25 °C, consistent with the reported rheological results and comparable to previously reported gel transition temperatures.^9^ Although implementing the reported potentials in computer simulations is beyond the scope of the present study, their analytical form is designed to capture interparticle forces and torques critical to colloidal gelation and to enable the ease of use in simulations.

## Conclusion

This work introduces a robust and reproducible colloidal model system for rheological studies, enabling the precise synthesis, measurement, and modeling of interparticle forces. Leveraging click-like chemistry ensures uniform grafting—a critical factor in achieving the thermoreversible liquid-to-solid transition. By systematically tuning the reaction conditions, particularly the concentration of the grafting agent, the grafting density—and consequently the gelation temperature—can be precisely controlled.

In this study, grafting density was primarily modulated via the concentration of octadecyl-alkynoate in the reaction mixture. However, other parameters, such as the temperature, offer additional control. Notably, the solubility of octadecyl-alkynoate in isopropanol changes significantly between 25 and 40 °C, suggesting that the thermal control during grafting could influence polymer conformation and grafting efficiency via changes in the radius of gyration. The platform is versatile and can accommodate a range of ligands—including tetradecyl, eicosanyl, PEG, and PEG–PCL—as well as different particle substrates such as borosilicate glass and polystyrene. Grafting onto glass surfaces is particularly advantageous for functionalizing rheological fixtures, improving experimental stability and control.

The resulting interparticle potential comprises steric repulsion, van der Waals attraction, and a temperature-dependent short-range attractive force. The latter cannot be explained by van der Waals interactions alone, indicating that the grafting agent interpenetration contributes significantly to the gelation behavior. This detailed understanding of interparticle interactions provides a strong basis for accurate simulations and mechanistic insights into thermoreversible colloidal gels. Altogether, this end-to-end system lays the groundwork for integrated experimental and computational studies of colloidal gelation, offering a versatile and tunable platform for probing the structure–property relationships that govern soft matter behavior.

## Supplementary Material



## Notes

Experimental and characterization details; ligand layer characterization; rheological measurements; NMR spectra of grafting agents; potential calculations; and extended theory for potential calculations (PDF)

## References

[1] Läuger J., Stettin H. (2016). Effects of instrument and fluid inertia in oscillatory
shear in rotational rheometers. J. Rheol..

[2] Renggli D., Alicke A., Ewoldt R. H., Vermant J. (2020). Operating windows for
oscillatory interfacial shear rheology. J. Rheol..

[3] Larson R. G., Wei Y. (2019). A review of thixotropy and its rheological modeling. J. Rheol..

[4] Mewis J., Wagner N. J. (2009). Thixotropy. Adv. Colloid Interface
Sci..

[5] Rueb C. J., Zukoski C. (1997). Viscoelastic properties
of colloidal gels. J. Rheol..

[6] Choi J., Rogers S. A. (2020). Optimal conditions for pre-shearing thixotropic or
aging soft materials. Rheol. Acta.

[7] Van
Helden A., Jansen J., Vrij A. (1981). Preparation and characterization
of spherical monodisperse silica dispersions in nonaqueous solvents. J. Colloid Interface Sci..

[8] Eberle A. P., Wagner N. J., Akgun B., Satija S. K. (2010). Temperature-dependent
nanostructure of an end-tethered octadecane brush in tetradecane and
nanoparticle phase behavior. Langmuir.

[9] Yin G., Solomon M. J. (2008). Soft glassy rheology model applied to stress relaxation
of a thermoreversible colloidal gel. J. Rheol..

[10] Hoekstra H., Mewis J., Narayanan T., Vermant J. (2005). Multi Length Scale
Analysis of the Microstructure in Sticky Sphere Dispersions during
Shear Flow. Langmuir.

[11] Eberle A. P., Castaneda-Priego R., Kim J. M., Wagner N. J. (2012). Dynamical arrest,
percolation, gelation, and glass formation in model nanoparticle dispersions
with thermoreversible adhesive interactions. Langmuir.

[12] Grant M. C., Russel W. B. (1993). Volume-fraction
dependence of elastic moduli and transition
temperatures for colloidal silica gels. Phys.
Rev. E: Stat. Phys., Plasmas, Fluids, Relat. Interdiscip. Top..

[13] Chen M., Russel W. B. (1991). Characteristics of flocculated silica
dispersions. J. Colloid Interface Sci..

[14] Varadan P., Solomon M. J. (2001). Shear-induced microstructural
evolution of a thermoreversible
colloidal gel. Langmuir.

[15] Sztucki M., Narayanan T., Belina G., Moussaïd A., Pignon F., Hoekstra H. (2006). Kinetic arrest and glass-glass transition
in short-ranged attractive colloids. Phys. Rev.
E: Stat., Nonlinear, Soft Matter Phys..

[16] Zanini M., Marschelke C., Anachkov S. E., Marini E., Synytska A., Isa L. (2017). Universal
emulsion stabilization from the arrested adsorption of
rough particles at liquid-liquid interfaces. Nat. Commun..

[17] Kuijk A., Van Blaaderen A., Imhof A. (2011). Synthesis of monodisperse,
rodlike
silica colloids with tunable aspect ratio. J.
Am. Chem. Soc..

[18] Müller F. J., Yang K., Isa L., Vermant J. (2025). Tuning particle aspect
ratio and surface roughness to modulate properties in colloidal gels. J. Colloid Interface Sci..

[19] Stöber W., Fink A., Bohn E. (1968). Controlled
growth of monodisperse
silica spheres in the micron size range. J.
Colloid Interface Sci..

[20] Ding S., Zhang C., Wei W., Qu X., Liu J., Yang Z. (2009). Amphiphilic patchy composite colloids. Macromol.
Rapid Commun..

[21] Maas M., Silverio C. C., Laube J., Rezwan K. (2017). Electrostatic assembly
of zwitterionic and amphiphilic supraparticles. J. Colloid Interface Sci..

[22] Müller F. J., Ramakrishna S. N., Isa L., Vermant J. (2025). Tuning Colloidal Gel
Properties: The Influence of Central and Noncentral Forces. Langmuir.

[23] Nabizadeh M., Nasirian F., Li X., Saraswat Y., Waheibi R., Hsiao L. C., Bi D., Ravandi B., Jamali S. (2024). Network physics
of attractive colloidal gels: Resilience, rigidity, and phase diagram. Proc. Natl. Acad. Sci. U.S.A..

[24] Hsiao L. C., Newman R. S., Glotzer S. C., Solomon M. J. (2012). Role of isostaticity
and load-bearing microstructure in the elasticity of yielded colloidal
gels. Proc. Natl. Acad. Sci. U.S.A..

[25] Varga Z., Swan J. W. (2018). Large scale anisotropies in sheared
colloidal gels. J. Rheol..

[26] Swan J. W., Brady J. F. (2007). Simulation of hydrodynamically interacting particles
near a no-slip boundary. Phys. Fluids.

[27] Varga Z., Wang G., Swan J. (2015). The hydrodynamics of colloidal gelation. Soft Matter.

[28] Colombo, G. Controlling Microstructure and Rheological Properties of Colloidal Gels. PhD Thesis, ETH Zurich, 2019.

[29] Johnson L. C., Zia R. N. (2021). Phase mechanics
of colloidal gels: osmotic pressure
drives non-equilibrium phase separation. Soft
Matter.

[30] Asakura S., Oosawa F. (1958). Interaction between
Particles Suspended in Solutions
of Macromolecules. J. Polym. Sci..

[31] Dibble C. J., Kogan M., Solomon M. J. (2006). Structure and dynamics
of colloidal
depletion gels: coincidence of transitions and heterogeneity. Phys. Rev. E: Stat., Nonlinear, Soft Matter Phys..

[32] Bevan M. A., Petris S. N., Chan D. Y. (2002). Solvent quality
dependent continuum
van der Waals attraction and phase behavior for colloids bearing nonuniform
adsorbed polymer layers. Langmuir.

[33] Flory, P. J. Principles of polymer chemistry; Cornell university press, 1953.

[34] Ninham B.
W., Parsegian V. A., Weiss G. H. (1970). On the macroscopic theory of temperature-dependent
van der Waals forces. J. Stat. Phys..

[35] Parsegian V. A., Ninham B. (1970). Temperature-dependent van der Waals forces. Biophys. J..

[36] Sen A., Anicich V., Arakelian T. (1992). Dielectric
constant of liquid alkanes
and hydrocarbon mixtures. J. Phys. D: Appl.
Phys..

[37] Jansen J., De Kruif C., Vrij A. (1986). Attractions
in sterically stabilized
silica dispersions: I. Theory of phase separation. J. Colloid Interface Sci..

[38] Fenton O. S., Andresen J. L., Paolini M., Langer R. (2018). beta-Aminoacrylate
Synthetic Hydrogels: Easily Accessible and Operationally Simple Biomaterials
Networks. Angew. Chem., Int. Ed. Engl..

[39] He B., Su H., Bai T., Wu Y., Li S., Gao M., Hu R., Zhao Z., Qin A., Ling J., Tang B. Z. (2017). Spontaneous
Amino-yne Click Polymerization: A Powerful Tool toward Regio- and
Stereospecific Poly­(beta-aminoacrylate)­s. J.
Am. Chem. Soc..

[40] You M., Zhang L., Gmür T. A., Zhang K., Zürcher S., Li W., Yuan G., Spencer N. D., Pei J. (2022). Impact of graft architecture
of PEGylated copolymers assembly on hydroxyapatite in the differential
regulation of initial cell and bacterial adhesion. Appl. Surf. Sci..

[41] Peng X., McKenna G. B. (2014). Comparison of the
physical aging behavior of a colloidal
glass after shear melting and concentration jumps. Phys. Rev. E: Stat., Nonlinear, Soft Matter Phys..

[42] Bonacci F., Chateau X., Furst E. M., Fusier J., Goyon J., Lemaitre A. (2020). Contact and macroscopic ageing in
colloidal suspensions. Nat. Mater..

[43] Colombo G., Massaro R., Coleman S., Läuger J., Puyvelde P. V., Vermant J. (2019). Ultrafast imaging of soft materials
during shear flow. Korea Aust. Rheol. J..

[44] Bevan M. A., Prieve D. C. (1999). Direct measurement
of retarded van der Waals attraction. Langmuir.

[45] Dagastine R. R., Bevan M., White L. R., Prieve D. C. (2004). Calculation of van
der Waals forces with diffuse coatings: Applications to roughness
and adsorbed polymers. J. Adhes..

[46] Massaro R., Colombo G., Van Puyvelde P., Vermant J. (2020). Viscoelastic cluster
densification in sheared colloidal gels. Soft
Matter.

[47] Ruckdeschel P., Kemnitzer T. W., Nutz F. A., Senker J., Retsch M. (2015). Hollow silica
sphere colloidal crystals: insights into calcination dependent thermal
transport. Nanoscale.

[48] Issa S., Cousin F., Bonnevide M., Gigmes D., Jestin J., Phan T. N. T. (2021). Poly­(ethylene oxide) grafted silica nanoparticles:
efficient routes of synthesis with associated colloidal stability. Soft Matter.

[49] Winter H. H., Chambon F. (1986). Analysis of Linear
Viscoelasticity of a Crosslinking
Polymer at the Gel Point. J. Rheol..

[50] Van
Helden A., Vrij A. (1980). Static light scattering of concentrated
silica dispersions in apolar solvents. J. Colloid
Interface Sci..

[51] Strong L., Whitesides G. M. (1988). Structures
of self-assembled monolayer films of organosulfur
compounds adsorbed on gold single crystals: electron diffraction studies. Langmuir.

