# Supplementary material for: Fabrication and Modeling of a Thermoreversible Modular Core–Shell Colloidal System
Source: Langmuir. 2025 Sep 24;41(39):26779–88. doi: 10.1021/acs.langmuir.5c03418 (PMC12509310; doi:10.1021/acs.langmuir.5c03418)
Supplement: Supplementary file 1 [file la5c03418_si_001.pdf]

Supplementary Information

Fabrication and Modeling of a Thermoreversible Modular Core–Shell Colloidal System

Florence J. Müller*[1,3], Alec J. Pellicciotti[2], Shivaprakash Ramakrishna[1], Lucio Isa[1], Michael A. Bevan[2], and Jan Vermant[1]

[1]Department of Materials, ETH Zurich, Vladimir-Prelog-Weg 5, 8093 Zurich, Switzerland
[2]Chemical & Biomolecular Engineering, Johns Hopkins University, Baltimore, MD 21218, United States

Corresponding author: `fmuller@caltech.edu`

[3]Current affiliation: Department of Chemical Engineering, California Institute of Technology, Pasadena, CA 91125, USA

S1 Synthesis method

S1.1 Grafting of octadecyl to the surface of silica particles using click-like chemistry

Octadecyl was grafted to the surface of silica particles in a multi-step approach (see main text Fig. 3). Silica particles can be synthesized through the well know and understood Stöber process [1] (see main text Fig. 3a), which produces particles with -OH surface groups. Different sizes of particles can be achieved by changing the concentration of H_2O and ammonium hydroxide [2, 3]. The particles can then be functionalized by adding Trimethoxy[3-(methylamino)propyl]silane (MAPTMS), to the Stöber solution without an additional cleaning step, yielding secondary amine functionalized silica particles (see main text Fig. 3b). This functionalization is quite similiar to the functionalization of silica particles with primary amines using (3-Aminopropyl)triethoxysilane (APTES) [4, 5, 6, 7].

S1.1.1 Octadecane-alkynoate functionalization and purification

Octadecanol can be functionalized with an alkynoate group through Fischer esterification (see main text Fig. 3c). The Fischer esterification is performed in toluene under reflux conditions using a Dean-Stark trap with propiolic acid as the functionalizing agent and toluenesulfonic acid-p monohydrate (pTsOH) as a catalyst. The optimal method to isolate the product from the octadecane-alkynoate from the surplus of propiolic acid and the pTsOH was to evaporate the toluene using a rotary evaporator, dissolve the product in isopropanol at 40°C, and add the solution to an iced water bath under stirring. The octadecane-alkynoate precipitates and can be filtered and dried. The quality of the functionalization was assessed through H-NMR (see Fig. S1), where the indicators for successful modification is a triplet for the alpha proton carbon (18 at 4.12 ppm) and the singlet for the triple bond (23 at 2.79 ppm). This reaction can be performed on a 10 g - 100 g scale, which allows for an efficient workflow, as 10 g of the octadecane-alkynoate grafting agent is enough to graft about 200 g of silica particles.

S1.2 Click-like amine-yne reaction to colloids

In literature, the amine-yne click-like reaction has been performed in aqueous media [8] or tetrahydrofuran [9]. For the grafting of octadecane-alkynoate to the secondary-amine functionalized particles, water cannot be used as a media, as octadecyl is hydrophobic. It was found that the reaction did not take place in ethanol, most likely because of a trans-esterification reaction between the ester of the alkynoate group and the OH-group of the ethanol. Performing the reaction in isopropanol at 40 °C did lead to a successful and homogeneous grafting. Heating of the reaction was necessary to ensure the solubility of the octadecane-alynoate in isopropanol. The time scale of the click-like reaction was investigated using H-NMR spectroscopy, by mixing the octadecane-alkynoate with MAPTMS, and measuring the spectrum at different time points (see Fig. S2). Here, we used the grafting agent used to functionalize the silica particles, as H-NMR cannot detect solid structures such as the functionalized silica particles. The reaction was performed in deuterated dimethyl sulfoxide. The reaction rate is expected to be different depending on the solvent and whether the secondary amine is attached to a solid, however, this experiment is an indication of the reaction mechanism. Fig. S2 shows the NMR spectrum for the octadecane-alkynoate and 5 min, 30 min, 1 h and 2 h after mixing. The

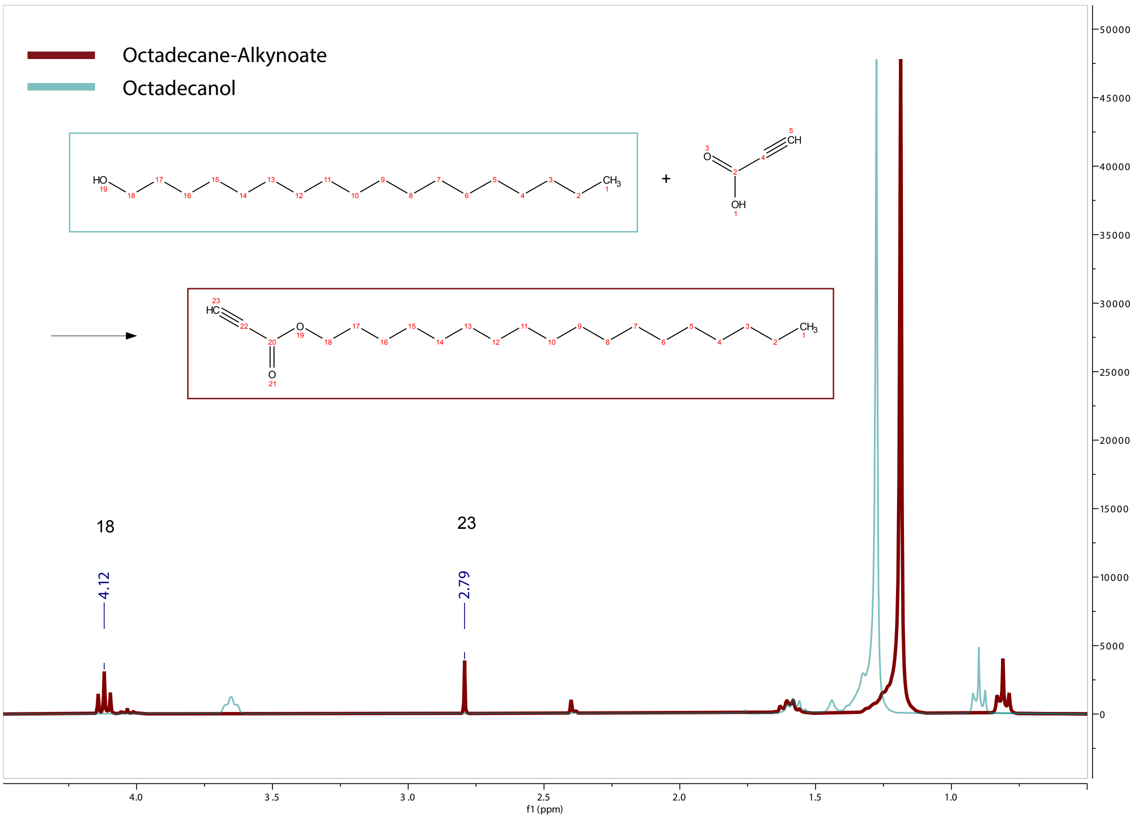

Figure S1: **NMR spectra of octadecanol (blue) and ocdtadecane-alkynoate (red)**, where the triplet at 4.12 ppm indicates the alpha-proton of the carbon chain at 2.79 ppm (carbon number 18 in the molecular structure) and the single peak indicates the the presence of the tripple bond (carbon number 23 in the molecular structure). The largest peaks at 1.2 ppm indicate the signal of the carbon chain, that remains the same for both samples.

single peak at 2.8 ppm represents the triple carbon bond, which reacts with the secondary amine, therefore the peak is decreasing over time and disappears after 2 h. The peak at 4.12 ppm represents the transformation from the secondary to the tertiary-amine. This experiment shows us that most of the reaction is likely to happen within the first hour after mixing, however, in practice, the reaction was left to stir for 4 h to ensure maximal grafting density.

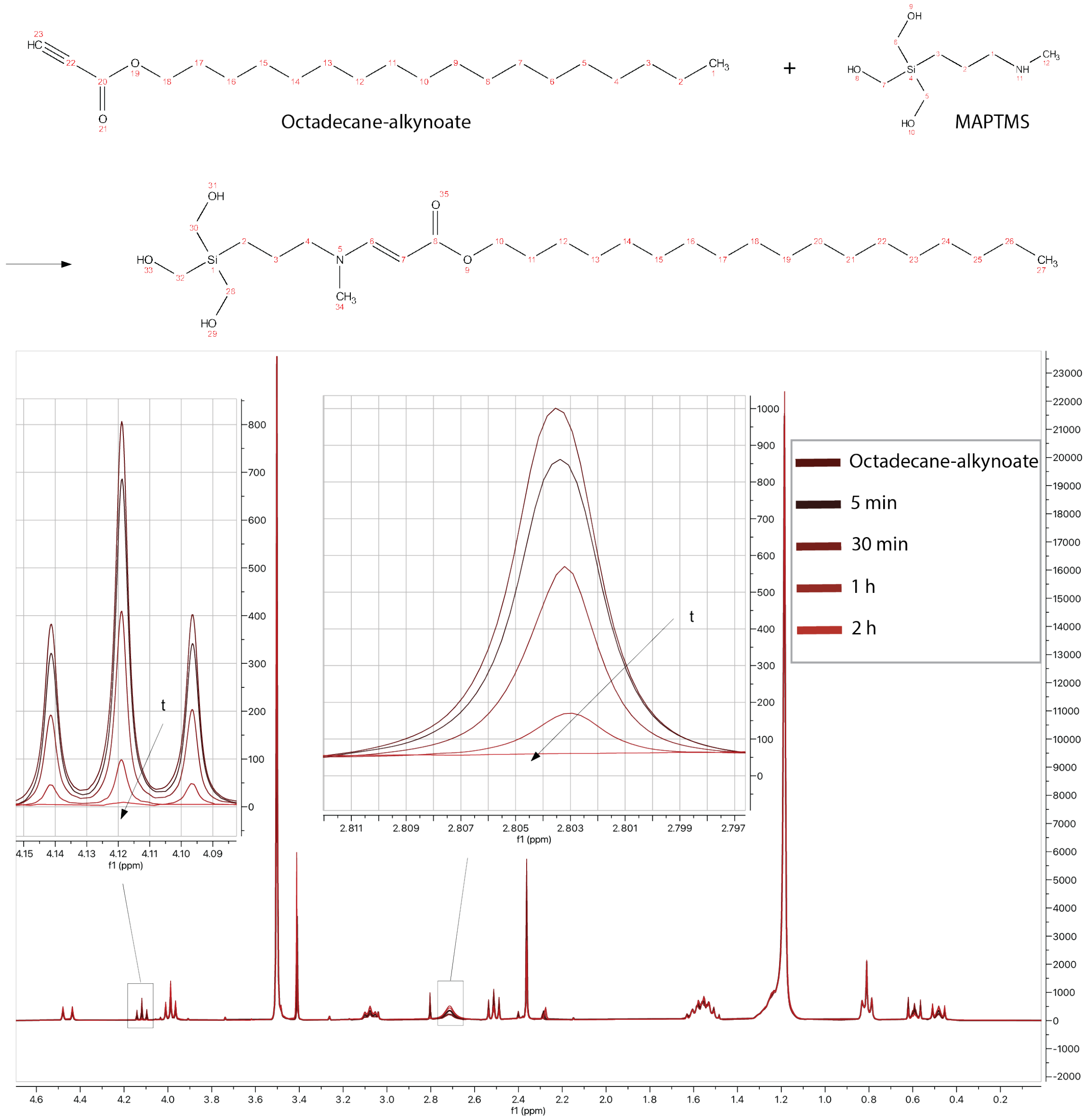

Figure S2: **NMR study of the click-like reaction over time using octadecane-alkynoate and MAPTMS** with the red shades getting lighter over time. The triplet at 4.12 ppm indicates the alpha proton (first carbon in the chain after the oxygen atom) which becomes smaller over time and eventually disappears. The singlet indicating the tripple bond, which undergoes the reaction with the secondary amine also becomes smaller over time and eventually disappears, showing that the octadecane-alkynoate fully reacts with the MAPTMS.

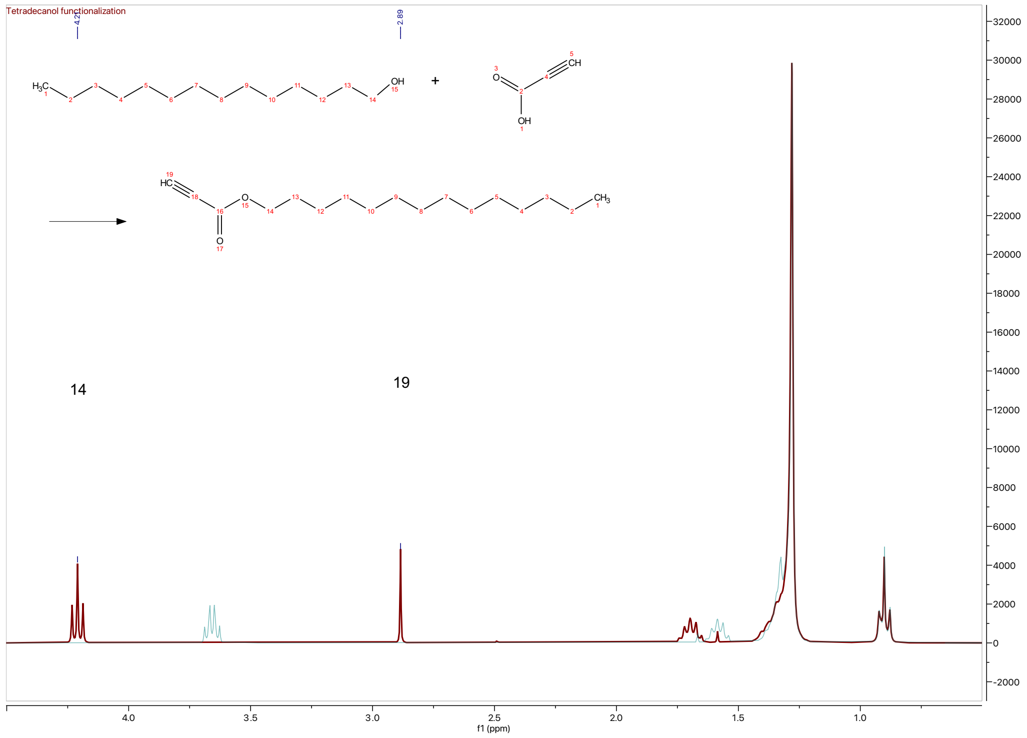

Figure S3: Alkynoate functionalization of teradecanol.

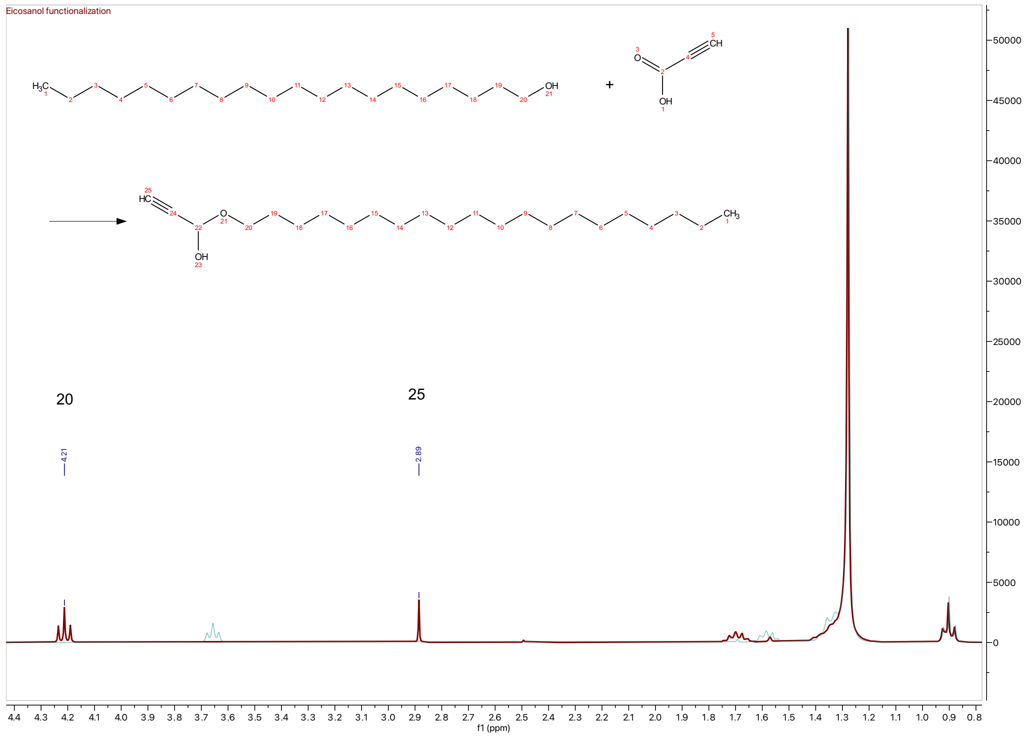

Figure S4: Alkynoate functionalization of eicosanol.

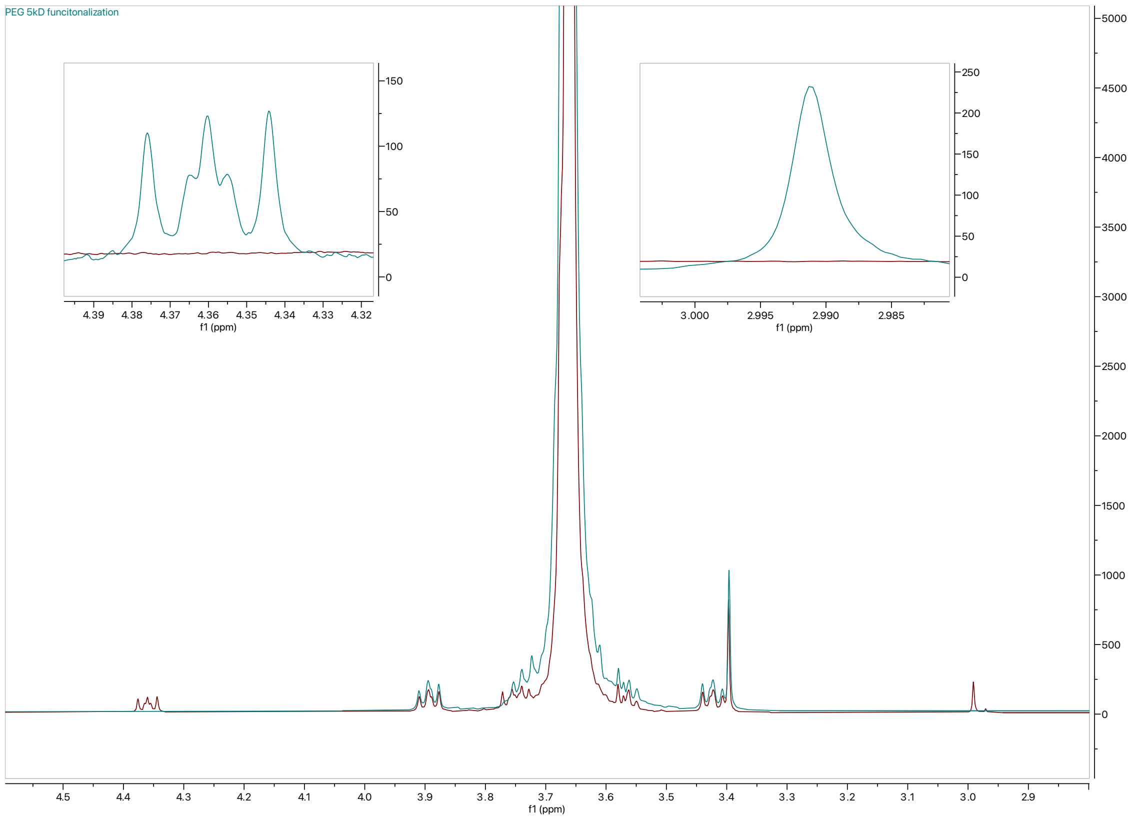

Figure S5: Alkynoate functionalization of methacrylated polyethylene glycol.

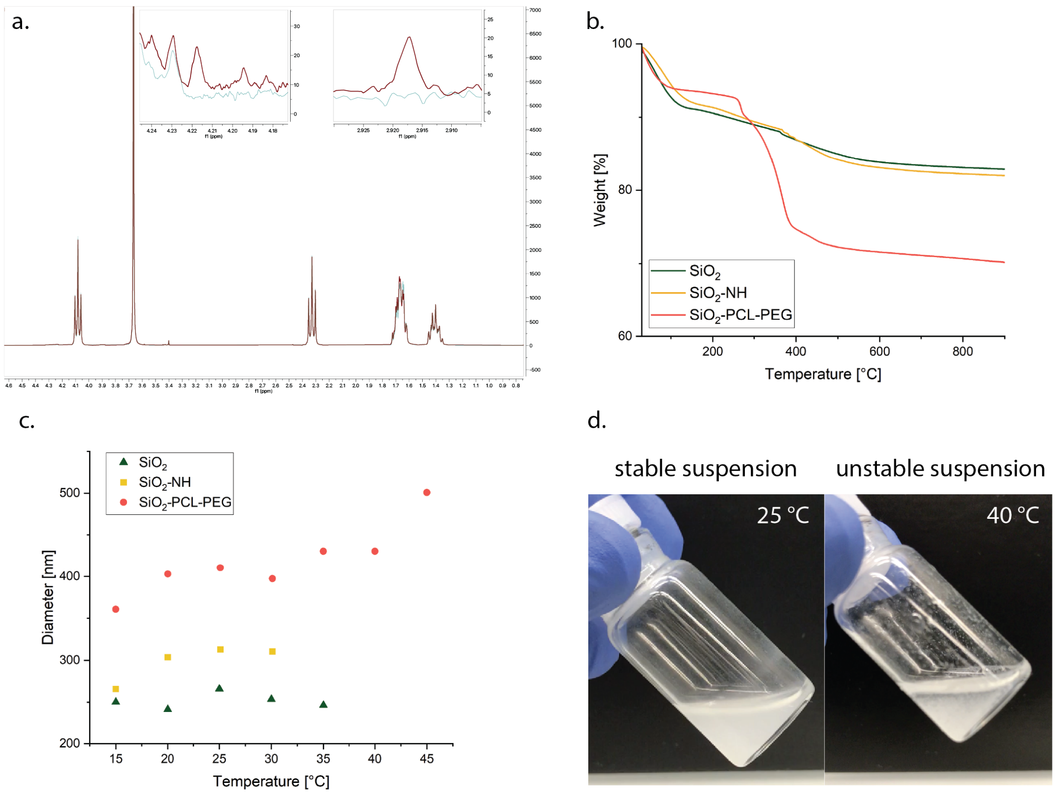

Figure S6: Functionalization and grafting of amphyphilic PEG-PCL to silica particles (300 nm in diamter) where the PCL attches to the silica and the PEG builds an outer corona. a. NMR of alkynoate grafted PEG-PCL, b. TGA proving successful grafting of the particles, c. DLS experiments showing successful grafting and temperature dependent swelling of the PCL layer, d. Image of the suspension that is stable at 25 °C and aggregates due to phobic interactions at 40 °C.

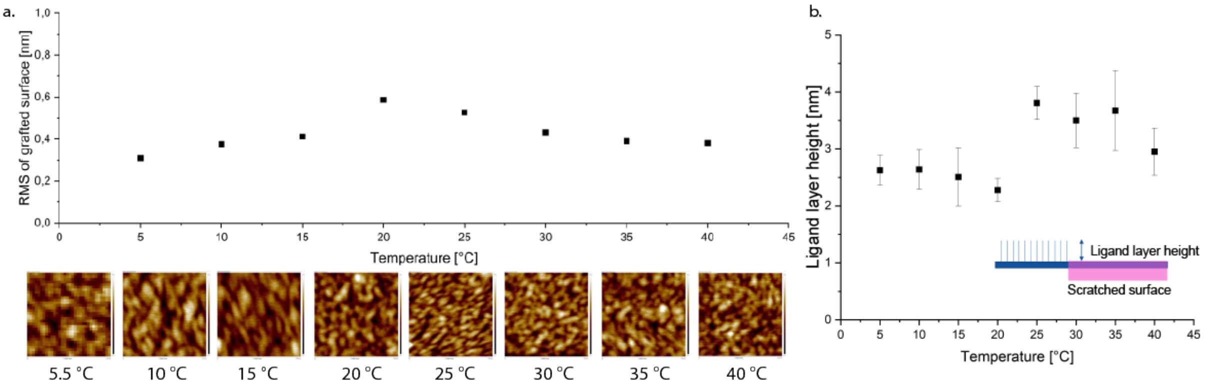

Figure S7: **AFM characterization of the octadecyl ligand layer as a function of temperature on a silicon wafer.** a. Surface morphology of the octadecyl layer, quantified using the root mean square roughness at different temperatures, the size of one image is (200 x 200 nm). b. Ligand layer thickness as a function of temperature

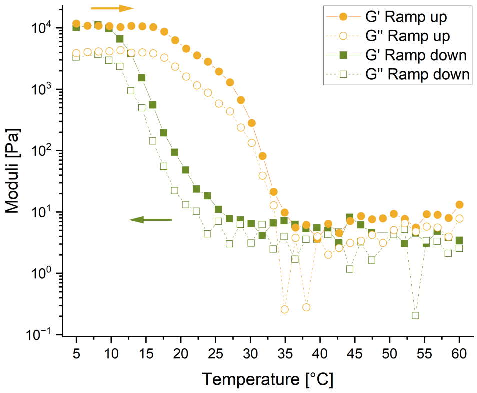

Figure S8: **Temperature hysteresis of the colloidal network formation.**

S2 TGA calculation

The mass of the secondary amine groups was calculated through

$$m_{MAPTMS} = m_{900}(SiO_2 - NH) - m_{900}(SiO_2 - OH), \tag{S1}$$

where $m_{900}(SiO_2 - NH)$ is the relative mass at 900°C of the control sample of amine grafted particles, and $m_{900}(SiO_2 - OH)$ are the control sample without any grafting, namely the pure Stöber particles. For all four cases of octadecyl grafted particles, the difference in relative mass loss due to the grafting layer can be calculated

$$m_{OD} = m_{900}(SiO_2 - OD) - m_{900}(SiO_2 - NH), \tag{S2}$$

where $m_{900}(SiO_2 - OD)$ is the relative mass of the samples with a grafted octadecyl layer. The number of moles of octadecyl grafted to the sample can then be calculated by dividing the mass by the molecular weight of the octadecyl-alkynoate

$$n_{OD} = \frac{m_{OD}}{MW_{OD-alkynoate}}. \tag{S3}$$

The total surface area of the particles was assessed by the initial weight of the sample over the mass of a single particle, which was calculated through the size and the density of a single particle, the latter of which was assumed to be 1.8 g/cm^3 [10]. The size of the particles in this experiment are 300 nm in diameter, which leads to a single particle surface A_{single} of 2.85·10^5 nm^2 a total surface area of

$$A_{tot} = \frac{100}{m_{single}} A_{single}, \tag{S4}$$

assuming that the calculations are based on a 100 g sample (for ease of calculation). Finally, the grafting density can be calculated by dividing the number of moles of octadecyl per sample by the total surface area of the particles, and multiply by Avogadro's number N_A

$$GD_{OD} = \frac{n_{OD}}{A_{tot}} N_A. \tag{S5}$$

Macroscopically, the temperature dependent state transition from liquid to gel, can be detected through an increase in storage (G′) and loss (G″) modulus. Figure S8 shows that, for a temperature ramp at 1°C/min there is a clear hysteresis behavior between a ramp up and ramp down of the temperature. This is most probably due to the temperature distribution inside the sample, that first needs to equilibrate for some time. If the temperature distribution inside the sample is homogeneous, however, the elastic and loss modulus can be controlled with temperature.

S3 Potential calculations

S3.1 Superpostition of potentials

The superposition of the forces in Eq. 1 (main text) which describe the thermoreversible nature of colloidal silica gels are derived from the superposition of the corresponding potentials:

$$U_{\text{tot}} = U_{\text{hw}} + U_{\text{vdw}} + U_{\text{cc}}, \tag{S6}$$

where the van der Waals attraction is modeled using the Derjaguin approximation for a particle interacting with a wall, which can further be approximated as a simple power law such that:

$$U_{\text{vdw}}(z) = \frac{a}{6}\int_z^\infty \frac{A(z)}{z} \approx -(Aa/6)z^{-p}, \tag{S7}$$

and the attractive interactions between octadecyl chains are accounted for by an additional potential, which are physically motivated by the interactions between overlapping ligands. The potential consists of an adjustable temperature-dependent free energy Λ, which is multiplied by an "included" volume equivalent to the amount of overlap between octadecyl chains for a given separation distance, z (equivalent to calculating the excluded volume for depletion).

$$\begin{aligned} U_{\text{cc}} &= \Lambda V^{\text{pw}}_{\text{overlap}} \\ &= \Lambda\pi[(4/3)\delta^3 + 4\delta^2 a - 4\delta a z + a z^2 - \delta z^2 - \delta z^2 + (z^3/3)] \end{aligned} \tag{S8}$$

where $V_{overlap}$ is the overlap volume of octadecyl-grafted chains between a sphere and a wall. For systems of colloidal suspensions where particle-particle interactions exist, the overlap volume is defined by the volume of overlapping spheres, which is:

$$V^{pp}_{\text{overlap}} = \pi[(4/3)(a+\delta)^3(1 - (3/4)4(a+\delta)^{-1} + (1/16)r^3(a+\delta)^{-3})] \tag{S9}$$

where $r = z + 2a$. Finally, the hard wall repulsion reflects the temperature transition observed in AFM measurements, where $\alpha = 0$ at T $<$ 20 °C and $\alpha = 2\delta$ at $T \geq 25$C

$$U_{\text{hw}}(z, T) = \begin{cases} 0 & z \geq \alpha \\ \infty & z < \alpha. \end{cases} \tag{S10}$$

S3.2 Lifshitz Theory for computing van der Waals attraction

The separation dependent Hamaker constant in Eq. 2 (main text) was calculated from the Lifshitz theory for half-spaces of the same material (1) separated by a medium (2) as [11]

$$A_{121}(l) = -\frac{3}{2}k_B T \sum_{n=0}^{\infty}{}' \int_{r_n}^{\infty} x\Big(\ln\left[1 - (\Delta_{12})^2 e^{-x}\right] + \ln\left[1 - (\bar{\Delta}_{12})^2 e^{-x}\right]\Big)\, dx \tag{S11}$$

$$\Delta_{\text{jk}} = \frac{\epsilon_j s_k - \epsilon_k s_j}{\epsilon_j s_k + \epsilon_k s_j} \qquad s_k^2 = x^2 + \left(\frac{2\xi_n l}{c}\right)^2 (\epsilon_k - \epsilon_2) \qquad \epsilon_k = \epsilon_k(i\xi_n)$$

$$\bar{\Delta}_{\text{jk}} = \frac{s_k - s_j}{s_k + s_j} \qquad r_n = \frac{2l\xi_n\sqrt{\epsilon_2}}{c} \qquad \xi_n = \frac{2\pi n k_B T}{\hbar}$$

For layered half-spaces which have symmetric homogenous coatings (3), Δ_{jk} in Eq. S11 is modified such that [11, 12]

$$\Delta_{21} = \frac{\Delta_{23} + \Delta_{31}\exp(-\delta s_3/l)}{1 + \Delta_{23} + \Delta_{31}\exp(-\delta s_3/l)}, \tag{S12}$$

where l is the separation between half-spaces or coatings ($z = l + \delta$). The dielectric response of the materials used in the investigated system is modeled as [13]

$$\epsilon_j(i\xi_n) = 1 + C_j/(1 + (\xi/\omega_j)^2) \text{ for } \xi_n \geq 0 \tag{S13}$$

where the material properties C_j and ω_j are defined in Table S1. Finally, given the solvation of tetradecane in the grafted octadecyl chains for the grafting density of the investigated system, the Clausius-Mossotti dielectric mixing formula is utilized to estimate the dielectric properties of the symmetric homogeneous coatings in the thermoreversible system using Eq. S14

$$\frac{\varepsilon(i\xi) - 1}{\varepsilon(i\xi) + 2} = \phi_{C_{18}}\frac{\varepsilon_{C_{18}}(i\xi) - 1}{\varepsilon_{C_{18}}(i\xi) + 2} + (1 - \phi_{C_{18}})\frac{\varepsilon_{C_{14}}(i\xi) - 1}{\varepsilon_{C_{14}}(i\xi) + 2} \tag{S14}$$

where ϕ is the volume fraction of the components. Two different Hamaker functions, $A(z)$, are computed for the system. At $z \geq 2\delta$, the Hamaker function is computed for half-spaces with symmetric homogeneous coatings across a medium (i.e. 1-4-2-4-1 using Material IDs in Table S1). At $z \leq 2\delta$, the Hamaker function is computed for half-spaces across a medium (i.e. 1-5-1 using Material IDs in Table S1).

Table S1: **Dielectric properties used in the Lifshitz theory to compute Hamaker functions.** [a]Bevan et al.[14]; [b]Hough and White[13]; [c]Extrapolation from trends in Cauchy plots[13]; [d]Eq. S14.

**Material**	**Material ID**	C_j	ω_j (10^{16} rad/s)
SiO_2 (silica)[a]	1	1.282	1.911
C_{14} (tetradecane)[b]	2	1.011	1.846
C_{18} (octadecane)[c]	3	1.058	1.837
60% C_{14}/40% C_{18}[d]	4	1.029	1.843
20% C_{14}/80% C_{18}[d]	5	1.049	1.839

S3.3 Particle-particle potentials and colloidal suspension stability

To ensure colloidal stability is recovered from the proposed model for bulk suspensions, the particle-particle van der Waals potential is modeled using the Hamaker approximation as [15]:

$$U_{\text{vdw}}^{pp}(z) = -\frac{A}{6}\left[\frac{2a^2}{z^2 - 4a^2} + \frac{2a^2}{z^2} + \ln\left(1 - \frac{4a^2}{z^2}\right)\right] \tag{S15}$$

which is also plotted in Fig. S9 and fits to a power law. At z = 5.9 nm (2 δ + 0.5 nm, which is an appropriate separation distance to approximate contact due to the breakdown of continuum mechanics in the Lifshitz theory [16]), the attraction due to van der Waals

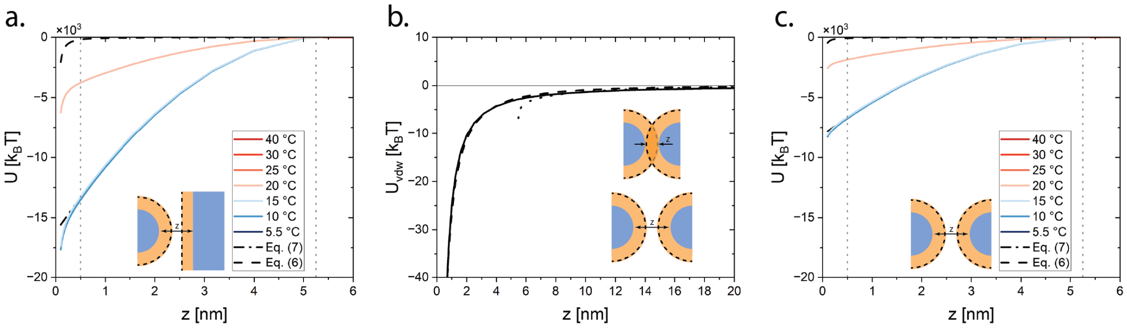

Figure S9: **Temperature-dependent superposition of potentials**(A) The individual components (Eq. (6) (–), Eq. (7) at T = 278 K (.-)) and the resulting superposition of hard wall, van der Waals, and chain-chain attraction potentials (Eq. (5)) for a sphere and a wall at 5.5, 10, 15, 20, 25, 30, and 40 °C. The derivative with respect to the silica-silica separation distance, z, yields the superposition of forces in Eq. (1). (B) the van der Waals potential computed from the Derjaguin approximation between two spheres using the Hamaker functions in Fig. 1A with a power law fit (black line). (C) The individual components and superposition of hard wall, van der Waals, and chain-chain attraction potentials for two spheres at 5.5, 10, 15, 20, 25, 30, and 40 °C, using the same plotting scheme as (A).

approximately -2.4 kT (Fig. S9b), which is insufficient to induce gelation. Therefore, the proposed model is also consistent with the stability behavior of bulk colloidal suspensions at temperatures greater than the transition of 25 °C.

References

[1] Werner Stöber, Arthur Fink, and Ernst Bohn. Controlled growth of monodisperse silica spheres in the micron size range. *Journal of colloid and interface science*, 26(1):62–69, 1968.

[2] Dmitry A Kurdyukov, Daniil A Eurov, Demid A Kirilenko, Vasily V Sokolov, and Valery G Golubev. Tailoring the size and microporosity of stöber silica particles. *Microporous and Mesoporous Materials*, 258:205–210, 2018.

[3] Rafael S Fernandes, Ivo M Raimundo Jr, and M Fernanda Pimentel. Revising the synthesis of stöber silica nanoparticles: A multivariate assessment study on the effects of reaction parameters on the particle size. *Colloids and Surfaces A: Physicochemical and Engineering Aspects*, 577:1–7, 2019.

[4] Michele Zanini, Chiao-Peng Hsu, Tommaso Magrini, Emanuele Marini, and Lucio Isa. Fabrication of rough colloids by heteroaggregation. *Colloids and Surfaces A: Physicochemical and Engineering Aspects*, 532:116–124, 2017.

[5] Leyre Gomez, Manuel Arruebo, Victor Sebastian, Laura Gutierrez, and Jesus Santamaria. Facile synthesis of sio2–au nanoshells in a three-stage microfluidic system. *Journal of Materials Chemistry*, 22(40), 2012.

[6] Nopphawan Phonthammachai, James CY Kah, Guo Jun, Colin JR Sheppard, Malini C Olivo, Subodh G Mhaisalkar, and Timothy J White. Synthesis of contiguous silica- gold core- shell structures: critical parameters and processes. *Langmuir*, 24(9):5109–5112, 2008.

[7] C. P. Hsu, J. Mandal, S. N. Ramakrishna, N. D. Spencer, and L. Isa. Exploring the roles of roughness, friction and adhesion in discontinuous shear thickening by means of thermo-responsive particles. *Nat Commun*, 12(1):1477, 2021.

[8] O. S. Fenton, J. L. Andresen, M. Paolini, and R. Langer. beta-aminoacrylate synthetic hydrogels: Easily accessible and operationally simple biomaterials networks. *Angew Chem Int Ed Engl*, 57(49):16026–16029, 2018.

[9] B. He, H. Su, T. Bai, Y. Wu, S. Li, M. Gao, R. Hu, Z. Zhao, A. Qin, J. Ling, and B. Z. Tang. Spontaneous amino-yne click polymerization: A powerful tool toward regio- and stereospecific poly(beta-aminoacrylate)s. *J Am Chem Soc*, 139(15):5437–5443, 2017.

[10] AK Van Helden, JW Jansen, and A Vrij. Preparation and characterization of spherical monodisperse silica dispersions in nonaqueous solvents. *Journal of colloid and interface science*, 81(2):354–368, 1981.

[11] Dennis C Prieve and William B Russel. Simplified predictions of hamaker constants from lifshitz theory. *Journal of Colloid and Interface Science*, 125(1):1–13, 1988.

[12] Raymond R Dagastine, Michael Bevan, Lee R White, and Dennis C Prieve. Calculation of van der waals forces with diffuse coatings: Applications to roughness and adsorbed polymers. *The Journal of Adhesion*, 80(5):365–394, 2004.

[13] David B Hough and Lee R White. The calculation of hamaker constants from liftshitz theory with applications to wetting phenomena. *Advances in Colloid and Interface Science*, 14(1):3–41, 1980.

[14] Michael A Bevan, Simon N Petris, and Derek YC Chan. Solvent quality dependent continuum van der waals attraction and phase behavior for colloids bearing nonuniform adsorbed polymer layers. *Langmuir*, 18(21):7845–7852, 2002.

[15] BA Pailthorpe and WB Russel. The retarded van der waals interaction between spheres. *Journal of colloid and interface science*, 89(2):563–566, 1982.

[16] Jacob N Israelachvili. *Intermolecular and surface forces*. Academic press, 2011.
